# *trans*-Bis(ethyl­enediamine)­bis­{2-[*N*-(2-hy­droxy­eth­yl)oxamoyl­amino]­benzoato}nickel(II)

**DOI:** 10.1107/S1600536810032848

**Published:** 2010-08-21

**Authors:** Jun Yu

**Affiliations:** a College of Plant Science, Tarim University, Xinjiang 843300, People’s Republic of China

## Abstract

The title mononuclear Ni^II^ complex, [Ni(C_11_H_11_N_2_O_5_)_2_(C_2_H_8_N_2_)_2_], is built up by inversion symmetry associated with the central Ni atom. The ethyl­enediamine ligands are non-planar. The r.m.s. deviation from the mean plane of the five-membered Ni–ethyl­amine chelate ring plane is 0.1945 Å. In the crystal structure, complex mol­ecules are linked to each other *via* N—H⋯O and O—-H⋯O hydrogen bonding through translation symmetry along the *b* and *c* axes, resulting in an extended supra­molecular network.

## Related literature

For background to oxamido compounds, see: Ruiz *et al.* (1999 ▶); Ojima & Nonoyama (1988 ▶). For related structures, see: Icbudak *et al.* (2003 ▶).
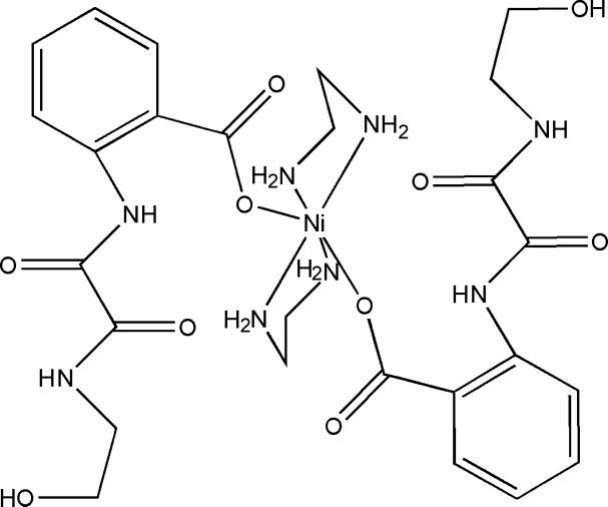


## Experimental

### 

#### Crystal data

[Ni(C_11_H_11_N_2_O_5_)_2_(C_2_H_8_N_2_)_2_]
*M* *_r_* = 681.35
Triclinic, 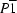

*a* = 8.266 (2) Å
*b* = 10.122 (3) Å
*c* = 10.260 (3) Å
α = 109.589 (3)°
β = 95.720 (3)°
γ = 103.788 (3)°
*V* = 770.1 (4) Å^3^
*Z* = 1
Mo *K*α radiation
μ = 0.70 mm^−1^
*T* = 298 K
0.36 × 0.35 × 0.32 mm

#### Data collection

Bruker SMART CCD diffractometer
Absorption correction: multi-scan (*SADABS*; Sheldrick, 1996 ▶) *T*_min_ = 0.845, *T*_max_ = 0.897
4112 measured reflections
2735 independent reflections
2385 reflections with *I* > 2σ(*I*)
*R*_int_ = 0.016

#### Refinement

*R*[*F* ^2^ > 2σ(*F* ^2^)] = 0.033
*wR*(*F* ^2^) = 0.084
*S* = 1.07
2735 reflections
206 parameters
H-atom parameters constrained
Δρ_max_ = 0.22 e Å^−3^
Δρ_min_ = −0.40 e Å^−3^

### 

Data collection: *SMART* (Bruker, 1998 ▶); cell refinement: *SAINT* (Bruker, 1998 ▶); data reduction: *SAINT*; program(s) used to solve structure: *SHELXS97* (Sheldrick, 2008 ▶); program(s) used to refine structure: *SHELXL97* (Sheldrick, 2008 ▶); molecular graphics: *SHELXTL* (Sheldrick, 2008 ▶); software used to prepare material for publication: *SHELXL97* and *PLATON* (Spek, 2009 ▶).

## Supplementary Material

Crystal structure: contains datablocks I, global. DOI: 10.1107/S1600536810032848/si2287sup1.cif

Structure factors: contains datablocks I. DOI: 10.1107/S1600536810032848/si2287Isup2.hkl

Additional supplementary materials:  crystallographic information; 3D view; checkCIF report

## Figures and Tables

**Table 1:** Selected bond lengths (Å)

Ni1—N3	2.0829 (18)
Ni1—N4	2.0847 (18)
Ni1—O1	2.1357 (14)

**Table 2:** Hydrogen-bond geometry (Å, °)

*D*—H⋯*A*	*D*—H	H⋯*A*	*D*⋯*A*	*D*—H⋯*A*
N3—H3*A*⋯O3^i^	0.90	2.22	2.976 (2)	142
N4—H4*B*⋯O2^ii^	0.90	2.30	3.001 (3)	134
O5—H5*A*⋯O2^iii^	0.82	1.94	2.727 (3)	160

Symmetry codes: (i) 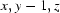; (ii) 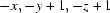; (iii) 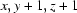.

**Fig. 1.**
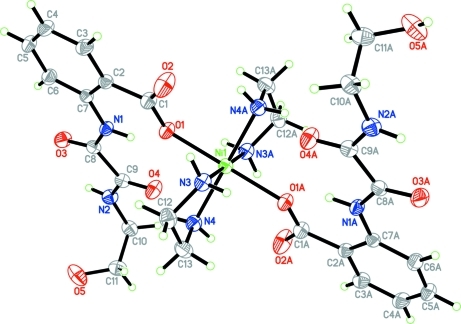
The molecular structure of (I) with 30% displacement ellipsoids. Inversion related atoms, labelled A, complete the metal complex with [Symmetry code ii = -x, -y + 1, -z + 1].

**Fig. 2.**
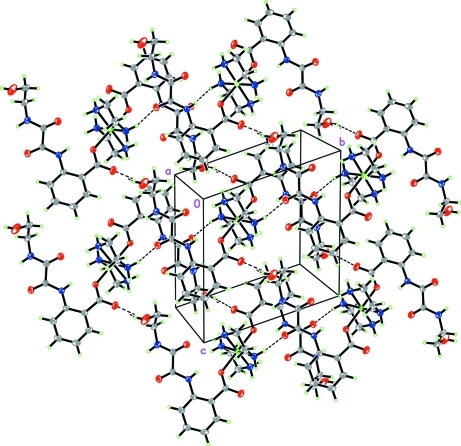
Packing diagram of (I). The hydrogen bonds are shown by the dashed lines.

## supplementary crystallographic information

### Comment

Oxamido compounds and their complexes have been investigated extensively (Ruiz *et al.*, 1999) by virtue of their bioactivities and the versatile bridging function (Ojima & Nonoyama, 1988). We selected 2-[N'-(ethanolamine)-oxamido]benzoate as a bridging ligand and ethylenediamine as another ligand to synthesize a new mononuclear nickel(II) compound, (I).

The title compound, (Fig. 1), is a mononuclear nickel(II) complex containing a total of 45 non-H atoms. The molecule is centrosymmetric with the central core Ni atom and the structure is similar to those seen previously in resemble compounds (Icbudak *et al.*, 2003). The Ni1 atom is in a trans-octahedral coordination geometry. Here, O1 and O1^ii^ [symmetry code: -*x*, -*y* + 1, -*z* + 1] are in axial positions [O1—Ni1—O1^ii^ =180.0°] and the N atoms of the two ethylenediamine groups are in equatorial positions. The sum of the equatorial N—Ni—N angles is 360.0°, indicating a coplanarity for these atoms. The planar oxamide group (r.m.s. deviation 0.0056 Å) displays a transoid conformation and makes a dihedral angle of 4.2 (8)° with the benzene ring (r.m.s. deviation 0.0031 Å), whereas the ethanol plane is rotated out of the oxamide group by a dihedral angle of 73.4 (8)°.

In the crystal structure, the mononuclear molecules are linked by the N-H···O and O-H···O intermolecular hydrogen bonds into a two-dimensonal network extending parallel to the bc plane (Figure 2).

### Experimental

To a stirred solution of N-benzyl-N'-(ethanolamine)oxamide (2mmol, 0.496g) in methanol(20ml), sodium ethoxide (0.136 g, 2mmol) and Ni(ClO_4_)_2_^.^6H_2_O (0.366g, 1mmol) was added. 10 min later, ethylenediamine (0.056 g, 1mmol) was added. The mixture was then stirred and heated at 323K for 6 h, then filtered. By slow evaporation of the filtrate, green crystals suitable for X-ray investigation were obtained after three weeks. Yield, 56%, analysis, calculated for C_26_H_38_N_8_O_10_Ni: C 45.83, H, 5.62; N 16.45%; found: C 45.81, H 5.68, N, 16.49%.

### Refinement

H atoms were positioned geometrically [0.93 (CH), 0.97 (CH_2_), 0.86 (NH), 0.90 (NH_2_) and 0.82 (OH)Å] and constrained to ride on their parent atoms with *U*_iso_(H) =1.2(1.5 for hydroxy O) *U*_eq_(C/N).

### Crystal data

**Table:**

[Ni(C_11_H_11_N_2_O_5_)_2_(C_2_H_8_N_2_)_2_]	*Z* = 1
*M**_r_* = 681.35	*F*(000) = 358
Triclinic, *P*1	*D*_x_ = 1.469 Mg m^−^^3^
Hall symbol: -P 1	Mo *K*α radiation, λ = 0.71073 Å
*a* = 8.266 (2) Å	Cell parameters from 2252 reflections
*b* = 10.122 (3) Å	θ = 2.6–27.0°
*c* = 10.260 (3) Å	µ = 0.70 mm^−^^1^
α = 109.589 (3)°	*T* = 298 K
β = 95.720 (3)°	Block, green
γ = 103.788 (3)°	0.36 × 0.35 × 0.32 mm
*V* = 770.1 (4) Å^3^	

### Data collection

**Table:**

Bruker SMART CCD diffractometer	2735 independent reflections
Radiation source: fine-focus sealed tube	2385 reflections with *I* > 2σ(*I*)
graphite	*R*_int_ = 0.016
φ and ω scans	θ_max_ = 25.2°, θ_min_ = 2.2°
Absorption correction: multi-scan (*SADABS*; Sheldrick, 1996)	*h* = −9→5
*T*_min_ = 0.845, *T*_max_ = 0.897	*k* = −12→12
4112 measured reflections	*l* = −12→12

### Refinement

**Table:**

Refinement on *F*^2^	Primary atom site location: structure-invariant direct methods
Least-squares matrix: full	Secondary atom site location: difference Fourier map
*R*[*F*^2^ > 2σ(*F*^2^)] = 0.033	Hydrogen site location: inferred from neighbouring sites
*wR*(*F*^2^) = 0.084	H-atom parameters constrained
*S* = 1.07	*w* = 1/[σ^2^(*F*_o_^2^) + (0.0403*P*)^2^ + 0.172*P*] where *P* = (*F*_o_^2^ + 2*F*_c_^2^)/3
2735 reflections	(Δ/σ)_max_ < 0.001
206 parameters	Δρ_max_ = 0.22 e Å^−^^3^
0 restraints	Δρ_min_ = −0.40 e Å^−^^3^

### Special details

**Table:**

Geometry. All esds (except the esd in the dihedral angle between two l.s. planes) are estimated using the full covariance matrix. The cell esds are taken into account individually in the estimation of esds in distances, angles and torsion angles; correlations between esds in cell parameters are only used when they are defined by crystal symmetry. An approximate (isotropic) treatment of cell esds is used for estimating esds involving l.s. planes.
Refinement. Refinement of F^2^ against ALL reflections. The weighted R-factor wR and goodness of fit S are based on F^2^, conventional R-factors R are based on F, with F set to zero for negative F^2^. The threshold expression of F^2^ > 2sigma(F^2^) is used only for calculating R-factors(gt) etc. and is not relevant to the choice of reflections for refinement. R-factors based on F^2^ are statistically about twice as large as those based on F, and R- factors based on ALL data will be even larger.

### Fractional atomic coordinates and isotropic or equivalent isotropic displacement parameters (Å^2^)

**Table:**

	*x*	*y*	*z*	*U*_iso_*/*U*_eq_	
Ni1	0.0000	0.5000	0.5000	0.03350 (13)	
O1	0.12334 (19)	0.66239 (15)	0.42423 (15)	0.0405 (4)	
O2	0.1944 (3)	0.52116 (18)	0.2362 (2)	0.0746 (6)	
O3	0.2826 (2)	1.19597 (16)	0.59663 (16)	0.0515 (4)	
O4	0.0627 (2)	0.92690 (17)	0.70836 (18)	0.0548 (4)	
O5	0.3278 (2)	1.3059 (2)	1.0935 (2)	0.0739 (6)	
H5A	0.3030	1.3824	1.1274	0.111*	
N1	0.2153 (2)	0.94573 (18)	0.49224 (17)	0.0349 (4)	
H1	0.1599	0.8678	0.5030	0.042*	
N2	0.1282 (2)	1.1741 (2)	0.80736 (19)	0.0435 (4)	
H2	0.1738	1.2541	0.7958	0.052*	
C1	0.1939 (3)	0.6434 (2)	0.3180 (2)	0.0411 (5)	
C2	0.2816 (2)	0.7770 (2)	0.2880 (2)	0.0353 (5)	
C3	0.3576 (3)	0.7547 (3)	0.1703 (2)	0.0451 (5)	
H3	0.3529	0.6597	0.1141	0.054*	
C4	0.4394 (3)	0.8697 (3)	0.1350 (3)	0.0493 (6)	
H4	0.4900	0.8525	0.0563	0.059*	
C5	0.4454 (3)	1.0099 (3)	0.2174 (3)	0.0501 (6)	
H5	0.4996	1.0878	0.1935	0.060*	
C6	0.3726 (3)	1.0370 (3)	0.3347 (2)	0.0442 (5)	
H6	0.3780	1.1328	0.3893	0.053*	
C7	0.2905 (2)	0.9221 (2)	0.3726 (2)	0.0335 (4)	
C8	0.2178 (3)	1.0721 (2)	0.5916 (2)	0.0358 (5)	
C9	0.1276 (3)	1.0487 (2)	0.7090 (2)	0.0376 (5)	
C10	0.0570 (3)	1.1848 (3)	0.9330 (2)	0.0462 (6)	
H10A	−0.0433	1.1021	0.9102	0.055*	
H10B	0.0221	1.2736	0.9637	0.055*	
C11	0.1807 (3)	1.1869 (3)	1.0509 (2)	0.0515 (6)	
H11A	0.1260	1.1908	1.1308	0.062*	
H11B	0.2125	1.0966	1.0210	0.062*	
N3	0.2112 (2)	0.42116 (19)	0.49185 (19)	0.0400 (4)	
H3A	0.1841	0.3313	0.4960	0.048*	
H3B	0.2471	0.4148	0.4106	0.048*	
N4	0.1301 (2)	0.63455 (19)	0.70438 (19)	0.0418 (4)	
H4A	0.1743	0.7267	0.7092	0.050*	
H4B	0.0590	0.6360	0.7655	0.050*	
C12	0.3456 (3)	0.5236 (3)	0.6128 (3)	0.0516 (6)	
H12A	0.4037	0.6070	0.5916	0.062*	
H12B	0.4281	0.4752	0.6321	0.062*	
C13	0.2663 (3)	0.5744 (3)	0.7395 (3)	0.0518 (6)	
H13A	0.2202	0.4928	0.7675	0.062*	
H13B	0.3519	0.6490	0.8178	0.062*	

### Atomic displacement parameters (Å^2^)

**Table:**

	*U*^11^	*U*^22^	*U*^33^	*U*^12^	*U*^13^	*U*^23^
Ni1	0.0382 (2)	0.0268 (2)	0.0349 (2)	0.01044 (15)	0.00982 (16)	0.00921 (16)
O1	0.0542 (9)	0.0298 (8)	0.0397 (8)	0.0123 (6)	0.0217 (7)	0.0119 (6)
O2	0.1148 (16)	0.0354 (10)	0.0756 (13)	0.0220 (10)	0.0614 (12)	0.0111 (9)
O3	0.0751 (12)	0.0310 (9)	0.0451 (9)	0.0113 (8)	0.0114 (8)	0.0131 (7)
O4	0.0739 (11)	0.0374 (9)	0.0573 (11)	0.0163 (8)	0.0340 (9)	0.0165 (8)
O5	0.0482 (10)	0.0763 (14)	0.0662 (13)	0.0182 (10)	0.0008 (9)	−0.0093 (11)
N1	0.0416 (10)	0.0286 (9)	0.0358 (9)	0.0091 (7)	0.0120 (8)	0.0132 (8)
N2	0.0546 (11)	0.0363 (10)	0.0374 (10)	0.0167 (8)	0.0110 (8)	0.0079 (8)
C1	0.0479 (13)	0.0354 (12)	0.0418 (13)	0.0143 (10)	0.0166 (10)	0.0129 (10)
C2	0.0339 (11)	0.0402 (12)	0.0334 (11)	0.0119 (9)	0.0093 (9)	0.0143 (9)
C3	0.0471 (13)	0.0517 (14)	0.0393 (12)	0.0180 (11)	0.0163 (10)	0.0157 (11)
C4	0.0446 (13)	0.0674 (17)	0.0418 (13)	0.0146 (11)	0.0195 (10)	0.0257 (12)
C5	0.0462 (13)	0.0561 (15)	0.0523 (15)	0.0053 (11)	0.0146 (11)	0.0305 (13)
C6	0.0473 (13)	0.0410 (13)	0.0451 (13)	0.0092 (10)	0.0116 (10)	0.0187 (11)
C7	0.0295 (10)	0.0390 (11)	0.0328 (11)	0.0092 (8)	0.0046 (8)	0.0151 (9)
C8	0.0414 (11)	0.0291 (11)	0.0349 (11)	0.0116 (9)	0.0007 (9)	0.0105 (9)
C9	0.0404 (12)	0.0361 (12)	0.0365 (12)	0.0158 (9)	0.0064 (9)	0.0108 (10)
C10	0.0445 (13)	0.0459 (13)	0.0408 (13)	0.0175 (10)	0.0102 (10)	0.0035 (10)
C11	0.0575 (15)	0.0591 (16)	0.0387 (13)	0.0267 (12)	0.0135 (11)	0.0113 (11)
N3	0.0437 (10)	0.0362 (10)	0.0447 (11)	0.0155 (8)	0.0149 (8)	0.0163 (8)
N4	0.0481 (11)	0.0346 (10)	0.0401 (10)	0.0108 (8)	0.0100 (8)	0.0111 (8)
C12	0.0397 (12)	0.0485 (14)	0.0643 (16)	0.0127 (11)	0.0071 (11)	0.0191 (12)
C13	0.0501 (14)	0.0504 (14)	0.0467 (14)	0.0111 (11)	−0.0024 (11)	0.0137 (12)

### Geometric parameters (Å, °)

**Table:**

Ni1—N3^i^	2.0829 (18)	C4—C5	1.373 (4)
Ni1—N3	2.0829 (18)	C4—H4	0.9300
Ni1—N4^i^	2.0847 (18)	C5—C6	1.374 (3)
Ni1—N4	2.0847 (18)	C5—H5	0.9300
Ni1—O1	2.1357 (14)	C6—C7	1.394 (3)
Ni1—O1^i^	2.1357 (14)	C6—H6	0.9300
O1—C1	1.267 (3)	C8—C9	1.533 (3)
O2—C1	1.242 (3)	C10—C11	1.495 (3)
O3—C8	1.222 (2)	C10—H10A	0.9700
O4—C9	1.219 (3)	C10—H10B	0.9700
O5—C11	1.401 (3)	C11—H11A	0.9700
O5—H5A	0.8200	C11—H11B	0.9700
N1—C8	1.336 (3)	N3—C12	1.471 (3)
N1—C7	1.406 (3)	N3—H3A	0.9000
N1—H1	0.8600	N3—H3B	0.9000
N2—C9	1.329 (3)	N4—C13	1.470 (3)
N2—C10	1.452 (3)	N4—H4A	0.9000
N2—H2	0.8600	N4—H4B	0.9000
C1—C2	1.518 (3)	C12—C13	1.505 (3)
C2—C3	1.392 (3)	C12—H12A	0.9700
C2—C7	1.413 (3)	C12—H12B	0.9700
C3—C4	1.379 (3)	C13—H13A	0.9700
C3—H3	0.9300	C13—H13B	0.9700
			
N3^i^—Ni1—N3	180.000 (1)	N1—C7—C2	118.73 (17)
N3^i^—Ni1—N4^i^	83.44 (7)	O3—C8—N1	127.5 (2)
N3—Ni1—N4^i^	96.56 (7)	O3—C8—C9	120.27 (19)
N3^i^—Ni1—N4	96.56 (7)	N1—C8—C9	112.28 (17)
N3—Ni1—N4	83.44 (7)	O4—C9—N2	125.4 (2)
N4^i^—Ni1—N4	180.000 (1)	O4—C9—C8	122.18 (19)
N3^i^—Ni1—O1	90.32 (6)	N2—C9—C8	112.47 (19)
N3—Ni1—O1	89.68 (6)	N2—C10—C11	112.25 (19)
N4^i^—Ni1—O1	90.28 (7)	N2—C10—H10A	109.2
N4—Ni1—O1	89.72 (7)	C11—C10—H10A	109.2
N3^i^—Ni1—O1^i^	89.68 (6)	N2—C10—H10B	109.2
N3—Ni1—O1^i^	90.32 (6)	C11—C10—H10B	109.2
N4^i^—Ni1—O1^i^	89.72 (7)	H10A—C10—H10B	107.9
N4—Ni1—O1^i^	90.28 (7)	O5—C11—C10	112.9 (2)
O1—Ni1—O1^i^	180.000 (1)	O5—C11—H11A	109.0
C1—O1—Ni1	127.60 (13)	C10—C11—H11A	109.0
C11—O5—H5A	109.5	O5—C11—H11B	109.0
C8—N1—C7	129.14 (17)	C10—C11—H11B	109.0
C8—N1—H1	115.4	H11A—C11—H11B	107.8
C7—N1—H1	115.4	C12—N3—Ni1	107.92 (14)
C9—N2—C10	124.2 (2)	C12—N3—H3A	110.1
C9—N2—H2	117.9	Ni1—N3—H3A	110.1
C10—N2—H2	117.9	C12—N3—H3B	110.1
O2—C1—O1	123.6 (2)	Ni1—N3—H3B	110.1
O2—C1—C2	118.0 (2)	H3A—N3—H3B	108.4
O1—C1—C2	118.39 (19)	C13—N4—Ni1	107.42 (14)
C3—C2—C7	118.33 (19)	C13—N4—H4A	110.2
C3—C2—C1	117.89 (19)	Ni1—N4—H4A	110.2
C7—C2—C1	123.78 (19)	C13—N4—H4B	110.2
C4—C3—C2	121.8 (2)	Ni1—N4—H4B	110.2
C4—C3—H3	119.1	H4A—N4—H4B	108.5
C2—C3—H3	119.1	N3—C12—C13	108.87 (18)
C5—C4—C3	119.2 (2)	N3—C12—H12A	109.9
C5—C4—H4	120.4	C13—C12—H12A	109.9
C3—C4—H4	120.4	N3—C12—H12B	109.9
C4—C5—C6	121.0 (2)	C13—C12—H12B	109.9
C4—C5—H5	119.5	H12A—C12—H12B	108.3
C6—C5—H5	119.5	N4—C13—C12	109.39 (19)
C5—C6—C7	120.5 (2)	N4—C13—H13A	109.8
C5—C6—H6	119.8	C12—C13—H13A	109.8
C7—C6—H6	119.8	N4—C13—H13B	109.8
C6—C7—N1	122.06 (19)	C12—C13—H13B	109.8
C6—C7—C2	119.2 (2)	H13A—C13—H13B	108.2
			
N3^i^—Ni1—O1—C1	−129.94 (18)	C7—N1—C8—O3	2.7 (4)
N3—Ni1—O1—C1	50.06 (18)	C7—N1—C8—C9	−176.75 (18)
N4^i^—Ni1—O1—C1	−46.50 (18)	C10—N2—C9—O4	2.7 (4)
N4—Ni1—O1—C1	133.50 (18)	C10—N2—C9—C8	−177.83 (18)
O1^i^—Ni1—O1—C1	46 (100)	O3—C8—C9—O4	−179.3 (2)
Ni1—O1—C1—O2	3.4 (3)	N1—C8—C9—O4	0.1 (3)
Ni1—O1—C1—C2	−177.07 (13)	O3—C8—C9—N2	1.2 (3)
O2—C1—C2—C3	0.0 (3)	N1—C8—C9—N2	−179.35 (18)
O1—C1—C2—C3	−179.48 (19)	C9—N2—C10—C11	86.0 (3)
O2—C1—C2—C7	−179.9 (2)	N2—C10—C11—O5	60.6 (3)
O1—C1—C2—C7	0.5 (3)	N3^i^—Ni1—N3—C12	−97 (100)
C7—C2—C3—C4	−0.2 (3)	N4^i^—Ni1—N3—C12	166.19 (14)
C1—C2—C3—C4	179.78 (19)	N4—Ni1—N3—C12	−13.81 (14)
C2—C3—C4—C5	−0.4 (4)	O1—Ni1—N3—C12	75.94 (14)
C3—C4—C5—C6	0.6 (4)	O1^i^—Ni1—N3—C12	−104.06 (14)
C4—C5—C6—C7	−0.1 (4)	N3^i^—Ni1—N4—C13	165.48 (14)
C5—C6—C7—N1	179.67 (19)	N3—Ni1—N4—C13	−14.52 (14)
C5—C6—C7—C2	−0.6 (3)	N4^i^—Ni1—N4—C13	3(100)
C8—N1—C7—C6	−5.3 (3)	O1—Ni1—N4—C13	−104.23 (15)
C8—N1—C7—C2	174.97 (19)	O1^i^—Ni1—N4—C13	75.77 (15)
C3—C2—C7—C6	0.8 (3)	Ni1—N3—C12—C13	39.4 (2)
C1—C2—C7—C6	−179.26 (19)	Ni1—N4—C13—C12	40.2 (2)
C3—C2—C7—N1	−179.53 (18)	N3—C12—C13—N4	−54.1 (2)
C1—C2—C7—N1	0.5 (3)		

Symmetry codes: (i) −*x*, −*y*+1, −*z*+1.

### Hydrogen-bond geometry (Å, °)

**Table:**

*D*—H···*A*	*D*—H	H···*A*	*D*···*A*	*D*—H···*A*
N3—H3A···O3^ii^	0.90	2.22	2.976 (2)	142.
N4—H4B···O2^i^	0.90	2.30	3.001 (3)	134.
O5—H5A···O2^iii^	0.82	1.94	2.727 (3)	160.

Symmetry codes: (ii) *x*, *y*−1, *z*; (i) −*x*, −*y*+1, −*z*+1; (iii) *x*, *y*+1, *z*+1.
